# Digitalis and Veratrum in the Treatment of Pneumonia*Read before the Austin District Medical Society, March 21, 1904.

**Published:** 1905-02

**Authors:** J. W. Carhart

**Affiliations:** Austin, Texas


For Texas Medical Journal.

### Digitalis and Veratrum in the Treatment of Pneumonia.*

BY J. W. CARHART, M. D., AUSTIN, TEXAS.

In presenting this subject for your consideration, it is not my purpose to either exhaust the theme or to dogmatize in the statements I shall make; or the propositions I shall lay down, but to elicit the truth, by a careful study, and a candid discussion of one of the most important subjects that could be brought to your attention today.

I wish to point out the fact that it goes without saying, or ought to, that there is not, and can not be, a fixed or stereotyped method of treating any disease known to the medical profession. As was remarked at a late meeting of the Travis County Medical Society, "Each disease must be treated on its own merits;" that is, all the circumstances, facts and conditions attending a case must be taken into account by the medical practitioner, not only in making a diagnosis, but in the selection, adaptation and administration of remedies. Treatment that would succeed in one case of pneumonia

*Read before the Austin District Medical Society, March 21, 1904.

would not succeed in all. "What is one man's meat is another man's poison."

At the same time there are certain prominent and fairly well established laws which should govern in all cases of treatment of a given disease; which make it possible, in the present conditions, to entitle medicine, in its broadest sense, to be classed under the head of science, though, as yet, far from being an *exact science.*

He who is not acquainted, in a general way, at least, with the pathology and pathogenesis of pneumonia, together with the therapeutical laws governing in its treatment, to such an extent as to be able to define, in his own mind, the conditions he has to deal with, and the nature, effects and dangers of the remedies he proposes to employ, so that he will not be governed by cast-iron rules to give heroic doses of digitalis or of aconite and veratrum from start to finish, is certainly not capable of treating a case of the disease here referred to.

Should any justification seem to be needed for the presentation of the subject of this paper, it is only necessary to refer to statements recently made by eminent practitioners of medicine, and by records of boards of health, that the mortality rate from pneumonia is increasing, not only in New York City, but throughout the entire country.

Ziemssen gives 50 per cent of deaths from pneumonia following measles. He lost one-half of his pneumonia patients under one year of age; two-fifths of the cases from one to three years, and one-fourth of those above three. The ratio of deaths from pneumonia among those of advanced years and enfeebled by age is even greater.

The disease prevails to an alarming extent in the State of Texas, particularly at certain seasons of the year; and as a consequence I have been led to make somewhat careful investigations into the nature and treatment of the disease and causes of the very large death rate amongst us, which, I have no doubt, will equal that reported by Ziemssen.

Whilst there are many factors contributing largely to swell the mortality record in this disease, such as insufficient clothing, uncomfortable dwellings, exposure in camp life, improper sanitation, insufficient nursing, bad habits as it regards the use of tobacco and alcoholics, I am persuaded that the use of digitalis in the first and second stages of the disease is responsible for the death of multitudes in the State of Texas.

There can be no approval, as it seems to me, of the administration of eight drops of a full-strength tincture of digitalis every three hours, from start to finish, in fifteen cases of alleged pneumonia, in which two of the number were old, and suffering from some undefined cardiac disease, notwithstanding all are alleged to have recovered.

A little calculation will show that 80 minims of a full-strength tincture of the drug was administered in each case in twenty-four hours, from start to finish, without reference to disturbance of gastric conditions or the cumulative effect of the drug. There is no authority on the subject with which I am acquainted that does not teach that such administration would be extremely toxic and deadly in its effects.

In order to a clear comprehension of our subject, it will be well for us, in the first place, to consider the physiological action of the drug in the condition of health.

Notwithstanding the conflicting opinions among writers on the drug there are several established facts upon which most, if not all, writers are agreed.

The National Dispensary, under "Digitalis," says: "Digitalis is given in doses of one to two grains, twice or thrice daily, until its effects begin to appear, *when it should be at once suspended.* The variable strength of digitalis requires that it should be used very cautiously, beginning with the minimum dose, which may be started at grain 1/60. This dose may be repeated at intervals of six or more hours, and gradually increased, but so as not to exceed grain 1/60 during twenty-four hours."

Professor Hobart Amory Hare, in his celebrated work on Therapeutics, p. 658, says: "The writer usually gives five drops of tincture of digitalis every eight hours, with five drops of tincture of belladonna every four hours." This is in his treatment of pneumonia. The belladonna is given to counteract the tendency of the digitalis to contract the capillaries.

From considerable experience with venesection in plethoric pneumonia patients during the stage of pulmonary engorgement, I am prepared to fully endorse the statement of Dr. Hare, in a late edition of his Therapeutics, p. 658, viz.: "Should the action of the heart become labored, the jugular veins distended and pulsating, and the radial pulse weak, while the face is cyanotic, then free venesection is to be practiced."

On the subject of bleeding in pneumonia, Osler, in the latest edition of his work, p. 135, says:

"The reproach of Von Helmont that 'A bloody Moloch presides in the chairs of medicine' can not be brought against this generation of physicians. Before Louis' iconoclastic paper on bleeding in pneumonia it would have been regarded as almost criminal to treat a case without venesection. We employ it nowadays much more than we did a few years ago, but more often late in the disease than early. To bleed at the very outset in robust, healthy individuals in whom the disease sets in with great intensity and high fever is, I believe, a good practice. I have seen instances in which it was very beneficial in relieving the pain and the dyspnea, reducing the temperature and allaying the cerebral symptoms.

Dr. Loomis, in Pepper's System of Medicine, holds that the lancet should be discarded in the treatment of pneumonia, because of the debilitating effect of the loss of blood, which views are not sustained by other eminent and careful observers, since venesection is practiced with plethoric patients, with rusty sputa, and other conditions pointed out by Hare; and for the further reason that the loss of four or five ounces of blood is inconsiderable in proportion, and is soon made up if plenty of water is allowed the patient.

Referring again to Hare, the same work, same page, he says: "It (venesection) will often save an apparently desperate case. Digitalis in the presence of this condition is not rapid enough in its effects."

I will now point out certain facts which in the pathological conditions of pneumonia contraindicate the use of digitalis, but indicate in the strongest manner the use of varatrum viride. But before doing so I undertake to say that, in view of the complicated and often contradictory action and conflicting effects of digitalis, and in view of the uncertainty of the strength of any tincture of digitalis, its use in pneumonia, particularly in what are known as the first and second stages, the use of the drug is attended with more danger, uncertainty, requiring more careful study, watchfulness and caution than any other drug in as common use in the entire materia medica; so that, to exhibit it at the rate of 80 minims in the twenty-four hours, the routine treatment in pneumonia cases, from start to finish, regardless of heart conditions, is nothing less than toxic and deadly.

Its cumulative effect must ever be borne in mind. Potter, in his Materia Medica, p. 303, says: "During the use of this drug (digitalis) for any length of time the muscle of the heart is so strained by over-stimulation that on suddenly assuming the upright position the cardiac energy may fail; more especially if the doses are administered too close together to admit of the elimination of one before the ingestion of the next. This is the explanation of the so-called, cumulative action of digitalis.

Another explanation is that it may stop its own excretion by arresting the renal circulation and the secretion of urine through extreme contraction of the renal vessels, and thus may really accumulate in the blood."

The following is from my paper on "Digitalis in the First and Second Stages of Pneumonia," read before the meeting of the American Medical Association at Washington, D. C., in 1891:

"Digitaline used hypodermically in frogs occasions tetanoid rigidity of the muscles, and an irregular, tonic contraction of the left ventricle of the heart, with slowness and unsteadiness of its rythm. The auricle does not seem to participate in this action of the ventricle, but becomes distended as the capacity of the ventricle diminishes. At the same time the pulse rate declines and the blood pressure increases."

Experimental investigation has demonstrated that large doses of digitaline produce marked contraction, and even a complete closure of the capillary vessels, a condition which necessarily involves an increased blood-pressure in the larger arteries. As we have before said, the auricle does not participate in the contraction affecting the ventricle, but becomes distended by the accumulated blood.

Digitalis is also held to act on the vaso-motor centers, thus producing contraction of the peripheral arterioles.

In small doses, digitalis and its preparations primarily increase pulse rate and tension, and if continued will lower the pulse rate without diminishing the tension. The supply of arterial blood is everywhere diminished under the influence of the drug, owing to the tonic contraction of both the left ventricle and of the arteries themselves.

All the heart muscles are not subject to contraction in an equal degree under the influence of digitalis; chiefly those of the left ventricle. It thus tends to obstruct cardiac circulation, and by preventing free passage of blood through the heart tends to produce death from syncope.

It certainly depresses the entire nervous system, if administered in sufficient doses to produce anything like its physiological action. It impairs digestion, diminishes urination, retards respiration, and interferes with nutrition of the heart itself.

Digitalis as an antipyretic and antiseptic is valueless. If any antiseptic effects should be produced by its exhibition they should be accompanied by disquieting results, and a tendency to heart-paralysis.

The result of various experiments proves that digitalis does not in the least modify favorably any acute inflammation, and especially pneumonia, pleurisy, and pericarditis; but that, on the contrary, it exposes the patient to the risk of sudden death.

Dr. T. J. Mays, in his recent work entitled, "Consumption, Pneumonia, and Their Kindred Allies," p. 472, says, speaking of therapeutical agents for the prevention of heart failure in pneumonia: "One of the best of these agents is strychnia. This drug, owing to its stimulating action on the nervous system in general, and the respiratory nerve supply in particular, is especially well adapted for use in this disease, as it is in fact in almost all affections of the pulmonary organs. Over and above this influence on the lungs, it is the equal of digitalis in enhancing the function of the heart, and in this manner it tends to overcome some of the most serious tendencies to death in this disease. But, in order to get the best effect of strychnine it must be given for tangible effects; that is, in doses large enough to approach the line of its toxic action, and for this reason it is useless when given in small doses."

Speaking of the use of digitalis in pneumonia, this author says: "With very large doses, such as are prescribed by some authorities, the author has had no experience."

Had he possessed the least confidence in the efficacy of large doses of digitalis in pneumonia, he doubtless would have had experience along that line.

It is now in order for us to get as clear an idea as possible of the pathological conditions in pneumonia, that we may understand the effects of digitalis in the first and second stages of this disease.

It will be necessary, for our present purpose, to discuss the fine distinctions that are made in this disease; such as bronchial pneumonia, catarrhal pneumonia, and lobular pneumonia. The term broncho-pneumonia will sufficiently indicate the form adapted to our purpose in this discussion.

The lungs are made up of bronchi, air passages, alveoli, pulmonary pleura and connective tissue stroma, containing blood vessels, lymphatics and nerves.

In pneumonia of the type under consideration the common course is for the inflammatory invasion to proceed from the larger to the smaller air-tubes, thence to advance into the finest, to the capillary bronchi, whence it communicates the inflammation to the terminal air passages and alveoli.

This process of invasion may be almost simultaneous and rapid, or it may take several days or weeks. The result, sooner or later, is involvement of the bronchi and more or less lobules and pulmonary hepitization, and possibly atelectasis.

I am aware that from a strictly pathological standpoint we may not speak of the stages of the disease as the stage of inflammation and engorgement, the stage of infiltration and red hepatization, as the result of engorgement; and the final or third stage of resolution or purulent infiltration, in favorable cases; or that of gray hepitization and possibly atelectasis premonitory of dissolution; and yet, clinically, this division would seem to be correct and very reasonable.

In order to a fuller understanding of our subject let us consider the condition and functions of the lungs in the first and second stages of the disease, and the resultant effect upon the circulatory apparatus.

In the early stages the bronchi contain more morbid secretions in the form of clear, viscid mucus, whilst in subsequent stages they are filled with creamy pus. There are sometimes found subpleural accumulations of somewhat inspisated, yellow secretions, contained in dilated alveoli, or in small globular dilatations of terminal bronchioles. They are probably caused by the secretion of particles of bronchial accumulations into the alveoli in the forcible inspiratory effect which follows paroxysms of coughing.

"The lung itself," says Pepper, "exhibits associated, in varying degrees, congestion, oedema, emphysema, collapse and pneumonic consolidation."

Jüergensen showed, more than thirty years ago, that there was stagnation in the lungs rather than an active hyperaemia, which results in the weakening of the heart. He also showed that the lung consolidation not only presented more or less obstruction to the pulmonary circulation, and hence necessitated increased action on the part of the right ventricle; but owing to the diminished respiratory surface this ventricle is obliged to do more work in order that the proper interchanges of gases may be effected in the lungs.

It is important for us now to consider the effect of the above conditions of the lungs on the heart and circulation, and we shall then be prepared to study the effects of digitalis in these cases.

It matters but little, so far as our present purpose is concerned, whether pneumonia be considered a systemic disease, with local manifestations in the lungs, or whether it be regarded as primarily affecting the lungs with systemic consequences.

We find, as a clinical and pathological fact, lungs engorged, swollen and hot, whatever may be the etiology, with blood statis, followed by hepatization and infiltration, and a consequent obstruction of circulation in the fine network of capillaries.

As a result of this condition of things we find a lack of aeration of the blood, with consequent carbonic acid poisoning of the whole system.

In consequence of the obstruction to the passage of blood through the lungs, the pulmonary vein is inadequately supplied, even with a partially aerated blood-stream. The blood coming to the left auricle and ventricle is hot in consequence of the extraordinary heat in the lungs. This hot, partially aerated blood-stream stimulates the heart to greater activity, whilst its nourishment furnished through the coronary arteries is inadequate to its overworked condition.

The longer this condition exists the more rapidly the heart will be overworked, as the irritability of the excito-motor ganglia is increased, and the tone of the vagus also reduced by the defective oxygen supply.

Owing to the back-pressure, so to speak, upon the venous blood-stream, from the right heart, caused by the blood statis in the lungs, we have extreme venous tension and intense strain upon the right heart.

In the outset of the disease we have a contracted condition of the capillaries, particularly of the periphery of the body, which adds to the venous tension. We now have labored respiration, owing to nature's effort to compensate for the restricted active lung area; we have a diminished and intensely inadequately aerated blood-stream from the lungs through the pulmonary vein to the left heart—we have a heart stimulated to frightful tension, in most cases, by the overworked blood, and inadequately nourished by vitiated blood through the coronary arteries—we have an inadequate deteriorated arterial supply, with capillary and venous engorgement.

With this condition of things we are told to give digitalis to relieve dyspnea and to strengthen the heart.

Dr. A. L. Loomis, speaking upon the subject of croupous pneumonia, says: "In all severe types of croupous pneumonia there are two prominent sources of danger: heat insufficiency and high temperature. There are, consequently, two prominent indications for treatment, viz.: to sustain the heart and reduce the temperature."

The same author further says: "Digitalis, of late years, has been extensively used to counteract heart-insufficiency; but it is very uncertain in its action in the heart-insufficiency of pneumonia, and has seemed to me more frequently to do harm than good. The nervous element of the heart failure contraindicates its use."

The foregoing wise words from Dr. Loomis must meet the unqualified endorsement of every intelligent physician.

In Nothnagel's Encyclopedia of Practical Medicine, p. 513, Dr. E. Aufrecht, speaking of the causes of mortality in pneumonia, says:

"The *causa proxima* of the fatal outcome in an overwhelming proportion of cases, is the gradual loss of power in the heart. 'Patients who die of pneumonia are killed by cardiac insufficiency,' says Dr. Jürgensen. This assumption he bases upon the following facts: (1) The pneumonia exudation increases the resistance in the lesser circulation and in this way throws an increased amount of work on the right ventricle; (2) the changes produced by pneumonia at and in the lung produce a diminution of the movement of the blood through these organs, especially as a part of the normal power of the circulation disappears and thus prevents the expansion of the sound lung, whereas just those parts of the surface of the lung which have been diminished by the pneumonic exudate requires greater power to force on the blood for the necessary interchange of gases to occur."

It is plainly to be seen that in this condition it is the height of folly to whip up an already overworked and enfeebled heart by the excessive use of digitalis, which contracts the blood vessels, thereby adding to the load that the failing heart has to carry, when relief might easily be obtained by methods that dilate the capillaries —relieve venous tension, and thus conserve the strength and integrity of the right heart or right auricle, and prevent loss of power in the heart. This can be effected by the judicious use of veratrum viride.

The use of alcohol in pneumonia and other fevers is condemned by these eminent German authorities.

We are told that digitalis reduces temperature, is a heart stimulant, slows and steadies the heart's action in pneumonia, and relieves dyspnoea, and, consequently, should be used, even in the stage of engorgement of this disease.

It is yet to be proven that it lowers temperature under any circumstances, except where it kills. It also remains to be proven that it slows and steadies the heart's action in the first and second stages of pneumonia, and that it relieves dyspnoea.

On the other hand, I affirm, and on the authority of eminent observers, coupled with my own experience, that in most cases in the first and second stages it increases dyspnoea, stimulates an already over-stimulated heart, renders the pulse unsteady and intermittent, as Dr. Loomis has said, tends to produce heart-paralysis, contracts the capillaries, thus adding to the blood stasis in the lungs, with increased venous tension and all its consequent train of evils. If pushed, in the condition to which I have referred, it will almost invariably produce death.

Veratria, with aconite, morphia and atropia, until the third stage commences, in most cases, slows the heart's action without depression; dilates the capillaries, thus relieving venous tension and the right heart, relieves dyspnoea, conserves the vital forces, reduces temperature, and lessens the inflammatory process.

If there is dicrotic pulse, especially from the use of digitalis, veratria, morphia, and atrophia will relieve, as I have witnessed in multitudes of cases.

These remedies should never be pushed to their unpleasant consequences, since the desired results can usually be obtained without.

